# Supplementary material for: Changes in the development of opioid tolerance on re-exposure among people who use heroin: A qualitative study
Source: PLoS One. 2022 Jun 23;17(6):e0269379. doi: 10.1371/journal.pone.0269379 (PMC9223324; doi:10.1371/journal.pone.0269379)
Supplement: S1 Appendix — (DOCX) [file pone.0269379.s001.docx]

**S1 Appendix. Interview topic guide**

**1. Introduction**

Explain purpose of research project

*The purpose of this study is to understand the views of people who use heroin on tolerance. You are the expert and I really appreciate you sharing your views with me.*

*We are interested in your views on tolerance to heroin rather than dependence or your experience with other drugs.*

Explain audio recording procedures

*Before we get started, I’d like to tell you that I will be recording the conversation to help us remember what we discussed. You can ask for the recording to be stopped at any time. What you say will be kept confidential and anonymous.*

Ensure participant has copy of participant information sheet

Answer any questions

Complete consent form

Commence audio recording

**2. Background and overview of drug practices**

To help us get an overview of the people we are speaking to, could you tell me your age please?

And how would you describe your current housing status?

What drugs have you used? (including alcohol)

What drugs are you *currently* using?

If heroin and crack, do you take these drugs together i.e. snowballing?

[ASK IF CURRENTLY USING HEROIN AND CRACK OR WHEN LAST USED IF DO NOT PROVIDE THIS INFORMATION]

Are you currently drinking alcohol?

Are you scripted? If so, what are you using (methadone, buprenorphine [Subutex])

When did you start taking heroin regularly?

How long have you been using OST (methadone, buprenorphine [Subutex]) for?

What method of using heroin did you start with? E.g. Inject, smoke (foil/in spill), rectally

Have you/did you changed the method of using heroin? E.g. Inject, smoke, rectally

[If yes] Why did you change?

Where do/did you use drugs? E.g. home, public

Who do/did you use drugs with? E.g. alone vs with others

PROMPTS/FOLLOW-ON QUESTIONS

Are there reasons for your choice of who you use with?

How many times have you been completely abstinent / clean?

How long have you been/did you remain abstinent for [in the past/at the moment]?

**3. Experience of tolerance following relapse/over time – **focus on tolerance rather than dependence****

What does ‘tolerance to drugs’ mean to you? / How would you describe ‘tolerance to drugs’?

Definition: “When drugs such as heroin are used repeatedly over time, tolerance may develop. Tolerance occurs when the person no longer responds to the drug in the way that person initially responded. Stated another way, it takes a higher dose of the drug to achieve the same level of response achieved initially.” (<https://www.drugabuse.gov/publications/teaching-packets/neurobiology-drug-addiction/section-iii-action-heroin-morphine/6-definition-tolerance>)

What is your personal experience of tolerance to heroin?

Have you experienced changes in physical signs and symptoms of tolerance (e.g. sickness, itchy and poor skin)?

Have you experienced changes in euphoria / effect from drug due to tolerance?

Have you experienced changes in your breathing slowing down (respiratory depression) due to tolerance?

When you began taking opiates regularly, do you remember how long it was before you started to notice you were developing tolerance?

If initially smoked heroin, did you experience any differences in tolerance development to when you moved on to injecting?

Following relapses of more than two weeks, do you remember how long it was before you started to notice you were developing tolerance?

How does your tolerance when you first started injecting regularly compare to after your most recent relapse of at least two weeks (e.g. experience of residual tolerance)?

What things do you think contribute to these changes in how tolerance develops following relapse?

How has your tolerance changed over time / after periods of abstinence?

- What things do you think influence how tolerance develops?
- Length of abstinence
- Poly-drug use/use of alcohol
- Environmental circumstances (e.g. where, when and with whom you use with)
- Strength of drugs/purity – i.e. has tolerance developed or is the strength of drugs weaker
- Mental health
- OST
- Do/did you do anything differently, in terms of how you use heroin if you become tolerant?
- Frequency of using
- Dose – does increasing dose stabilise/plateau
- Poly-drug use/use of alcohol to enhance effects of heroin
- Administration – smoking vs injecting
- Change type of drug / taking another drug as well as heroin

What is your personal experience of tolerance to OST (methadone, buprenorphine [Subutex])?

**4. Experience of abstinence**

*I’d like to ask you a couple of questions about your experience of being abstinent from OST and heroin for at least two weeks.*

What drug and/or OST were you using before you detoxed and became abstinent/clean (Subutex and Methadone – not expected to experience faster tolerance after relapse compared to detoxing from heroin alone)?

How has your experience of detoxing/becoming abstinent changed over time / over time (across a number of different detox experiences)? E.g. the way you do it and how it has felt?

**5. Experience of [re]lapse**

How did you start using after [re]lapse?

Changes in using behaviour (what, how, how much (use more or less), when, where, with whom) following period of abstinence?

What are your reasons for making these changes? (e.g. confidence, purity of drug)

When you relapsed after a period of more than 2 weeks did you use different drugs as well as heroin compared to when you first injecting heroin regularly (unless smoked both at beginning and after relapse – ask about smoking in this case)?

What role or influence did alcohol consumption have in relapse experience?

How does experience of ‘high’ change after relapse? (e.g. good/not as good)

How does this change by length of time detoxed – short, medium, long term?

**6. Experience of overdose following [re]lapse**

How does experience of your breathing slowing down (respiratory depression) or danger of overdose/going over change after detox and relapse?

Can you tell me about any things you do to avoid overdose when relapsing? (e.g. starting low)

Can you tell me about any experiences of overdose you’ve had when you relapsed?

- Were you aware of your breathing slowing down?
- If yes, have you experienced changes in your breathing slowing down (respiratory depression) due to tolerance?

What things do you think led to overdose following relapse?

If have not overdosed following relapse, why you do you think that is?

**7. Close**

That is the end of my questions. Before we finish is there anything I haven’t covered today that you would like to add?

End of audio recording

Thanks for participating

Provide £10 as a thank you for their time.
